# Genotype Distribution of Human Papillomavirus among Women with Cervical Cytological Abnormalities or Invasive Squamous Cell Carcinoma in a High-Incidence Area of Esophageal Carcinoma in China

**DOI:** 10.1155/2016/1256384

**Published:** 2016-08-17

**Authors:** Yuanyuan Wang, Shaohong Wang, Jinhui Shen, Yanyan Peng, Lechuan Chen, Ruiqin Mai, Guohong Zhang

**Affiliations:** ^1^Department of Pathology, Shantou Central Hospital and the Affiliated Shantou Hospital of Sun Yat-Sen University, Shantou, Guangdong 515041, China; ^2^Department of Laboratory Medicine, Shantou Central Hospital and the Affiliated Shantou Hospital of Sun Yat-Sen University, Shantou, Guangdong 515041, China; ^3^Department of Laboratory Medicine, The First Affiliated Hospital of Shantou University Medical College, Shantou, Guangdong 515041, China; ^4^Department of Pathology, Shantou University Medical College, Shantou, Guangdong 515041, China

## Abstract

Data of HPV genotype including 16 high-risk HPV (HR-HPV) and 4 low-risk HPV from 38,397 women with normal cytology, 1341 women with cervical cytology abnormalities, and 223 women with ISCC were retrospectively evaluated by a hospital-based study. The prevalence of high-risk HPV (HR-HPV) was 6.51%, 41.83%, and 96.86% in women with normal cytology, cervical cytology abnormalities, and ISCC, respectively. The three most common HPV types were HPV-52 (1.76%), HPV-16 (1.28%), and HPV-58 (0.97%) in women with normal cytology, whereas the most prevalent HPV type was HPV-16 (16.85%), followed by HPV-52 (9.55%) and HPV-58 (7.83%) in women with cervical cytology abnormalities. Specifically, HPV-16 had the highest frequency in ASC-H (24.16%, 36/149) and HSIL (35.71%, 110/308), while HPV-52 was the most common type in ASC-US (8.28%, 53/640) and LSIL (16.80%, 41/244). HPV-16 (75.78%), HPV18 (10.31%), and HPV58 (9.87%) were the most common types in women with ISCC. These data might contribute to increasing the knowledge of HPV epidemiology and providing the guide for vaccine selection for women in Shantou.

## 1. Introduction

Although liquid-based cytology screening for cervical cancer is recommended for women in most countries, however, cervical cancer accounts for 9% of the total new cancer cases and 8% of the total cancer deaths among females, with 85% especially in developing countries [1]. The crude incidence of cervical cancer was estimated to be about 8.7–11.3/100,000 in females in China, of whom 45.0% of cases died [2–4]. Human papillomavirus (HPV) infection is causally associated with cervical carcinogenesis. HPV-16 (4.82%), HPV-52 (4.52%), and HPV-58 (2.74%) represented the most prevalent high-risk HPV types of the nationwide prevalence of HPV infection in the general population in China [5]. In our previous study, we described that HPV-52 (4.07%), HPV-16 (3.63%), and HPV-58 (2.46%) were the most common types for women with normal cytology, while HPV-58 (14.12%), HPV-16 (13.72%), and HPV-52 (12.72%) were the most common types for women with cervical cytological abnormalities in Shantou's population [6]. However, no cervical cancer had been included in our previous study, and there is still no good characterization of HPV infection in women with normal cytology, cervical cytological abnormalities, and carcinoma in Shantou's population. Therefore, another independent cohort including normal cytology, cytological abnormalities, and cervical carcinoma is required to confirm whether HPV-58, HPV-16, and HPV-52 were the most frequent HPV types that have a substantial impact on cervical cancer in Shantou's population.

Recently, 9-valent virus-like particle vaccine against HPV, including HPV-6, HPV-11, HPV-16, and HPV-18 and five additional oncogenic types HPV-31, HPV-33, HPV-45, HPV-52, and HPV-58, has been developed [7]. Using this panel, targeting HPV-31/HPV-33/HPV-45/HPV-52/HPV-58 prevented an additional 4.2%–18.3% in USA and 12–19% of cervical cancers across four countries, that is, Brazil, Mexico, India, and China [8]. Therefore, understanding the HPV type distribution is important for tailoring regional screening programs. Furthermore, describing the baseline HPV type distribution before the introduction of vaccination programs facilitates further evaluation of potential impact of HPV vaccines in Shantou's population.

The purpose of this present study was to confirm the description of HPV prevalence and type-specific distribution in cervical cytological abnormalities in Shantou's population and to identify the most frequent multiple HPV types both in cervical cytological abnormalities and in invasive squamous cell carcinoma (ISCC).

## 2. Method and Material

### 2.1. Study Design and Population

This is a cross-sectional and retrospective study, which was composed of three kinds of consecutive participants who proceeded to routine screening from the Healthcare Center, who were referred for opportunistic screening and for evaluation of HPV-associated lesions from the Outpatient and Inpatient Gynaecological Clinic of the Shantou Central Hospital between January 2011 and April 2014. Participants who met the following criteria were included: participants who were permanent residents of Chaoshan area, were not pregnant, had not undergone hysterectomy, had no history of cervical surgery, and had never had pelvic radiation therapy. All participants included in the study gave their written informed consent. This study was approved by Research Ethics Boards at Shantou Central Hospital.

### 2.2. Cervical Specimen Collection and HPV DNA Extraction

In this study, Pap test based cervical specimens were collected from exfoliated cell. Cervical exfoliated cell specimens were obtained by a gynecologist as part of routine investigative procedures. Two separated cervical exfoliated cell specimens were collected independently for liquid-based cytological diagnosis and HPV DNA genotyping assays, respectively. Cervical slides were prepared using a liquid-based cytology method. Cytological classifications of disease grade were performed according to the Bethesda 2001 criteria (TBS2001) [9], including atypical squamous cells that cannot exclude high-grade squamous intraepithelial lesion (ASC-H), atypical squamous cells of undetermined significance (ASC-US), low-grade squamous intraepithelial lesion (LSIL), and high-grade squamous intraepithelial lesion (HSIL). However, ISCC information was obtained from routine histopathological diagnosis of biopsied tissue by an experienced pathologist. For HPV DNA extraction, exfoliated cells were stored at a specimen transport medium (Hybribio Biotechnology Limited Corp., Chaozhou, China). High-quality DNA was yielded from lysis of cells according to the manufacturer's instruction (Hybribio Biotechnology Limited Corp., Chaozhou, China).

### 2.3. HPV Genotyping

HPV genotyping was performed as described previously [6]; HPV DNA was amplified using KP-TC48 (Chaozhou Hybribio Biotechnology, China); and genotyping for HPV was performed by flow-through hybridization and gene chip by HybriMax (Chaozhou Hybribio Limited Corporation, Chaozhou, China). The chip covered 20 HPV genotypes: 15 high-risk (HR) types (HPV-16, HPV-18, HPV-31, HPV-33, HPV-35, HPV-39, HPV-45, HPV-51, HPV-52, HPV-53, HPV-56, HPV-58, HPV-59, HPV-66, and HPV-68) and 5 low-risk (LR) types (HPV-6, HPV-11, HPV-42, HPV-43, and HPV-44). Specifically, according to the International Agency for Research on Cancer (IARC) classification, group 1 (HPV-16, HPV-18, HPV-31, HPV-33, HPV-35, HPV-39, HPV-45, HPV-51, HPV-52, HPV-56, HPV-58, and HPV-59) were cancerogenic for humans and groups 2a (HPV-68) and 2b (HPV-26, HPV-53, HPV-66, HPV-73, and HPV-82) are probably cancerogenic [10]; therefore, our HR types include all the 12 types in group 1, HPV-68 in group 2a, and HPV-66 and HPV-53 in group 2b. The hybridized DNA fragments on the chip were detected via direct visualization of colorimetric changes.

### 2.4. Statistical Analysis

Categorical variables were summarized by absolute frequencies and percentages, and continuous variables were presented by means and Standard Deviation (SD). The Chi-square test and Fisher's exact test were used to compare proportions; the *t*-test was used to compare continuous variables among different groups. The data were analyzed by using SPSS 16 software (IBM SPSS Statistics, Armonk, NY, USA).

## 3. Results

### 3.1. HPV Prevalence and Type-Specific Distribution in the Study Overall Population

Combining cervical cytological and HPV-DNA testing including all of the 20 different HPV types was performed in a total of 39738 women. The mean age of those women was 38.16 ± 10.28 yrs. The prevalence of HPV infection and the distribution of the different HPV genotypes were shown in Tables 1 and 2. The prevalence of HPV in overall population and that in women with normal cytology were 8.30% (3300/39738) and 7.09% (2722/38397), respectively. The prevalence of HR-HPV was 8.30% (3300/39738) and 6.51% (2499/38397) in overall population and women with normal cytology as well. The most prevalent type was HPV-52 (2.04%, 809/39738) in overall population, followed by HPV-16 (1.81%, 718/39738) and HPV-58 (1.21%, 481/39738), and accounted for 24.52% (809/3300), 21.76% (718/3300), and 14.58% (481/3300) of HPV-positive women. The three most common HPV types were HPV-52 (1.76%, 681/38397), HPV-16 (1.28%, 492/38397), and HPV-58 (0.97%, 376/38397) in women with normal cytology.

### 3.2. HPV Type-Specific Distribution according to Cervical Cytology Abnormalities and ISCC

The 1341 (3.37%, 1341/39738) women with cervical cytology abnormalities were classified, of whom 11.11% (149/1341) had ASC-H, 47.73% (640/1341) had ASC-US, 22.97% (308/1341) had HSIL, and 18.20% (244/1341) had LSIL (Table 3). The mean age of those women was 41.54 ± 8.48 yrs. In the whole population with cervical cytology abnormalities included in our study, 41.83% (561/1341) were positive for HR-HPV, and, by lesion type, there were 48.32% (72/149) with ASC-H, 29.69% (190/640) with ASCU-US, 56.1% (173/308) with HSIL, and 51.64% (126/244) with LSIL. HR-HPV was greater in SIL (54.17%, 299/552) than in ASC (33.21%, 262/789, *P* < 0.001). The most prevalent HPV type was HPV-16 (16.85%, 226/1341) in all of the cervical cytology abnormalities, followed by HPV-52 (9.55%, 128/1341) and HPV-58 (7.83%, 105/1341) (Table 4). HPV-16, HPV-52, and HPV-58 accounted for 34.23% (459/1341) of all cervical cytology abnormalities. Specifically, HPV-16 had the highest frequency in ASC-H (24.16%, 36/149) and HSIL (35.71%, 110/308), while HPV-52 was the most common type in ASC-US (8.28%, 53/640) and LSIL (16.80%, 41/244).

A total of 223 ISCC were identified with mean age of 47.82 ± 6.79 yrs. The overall prevalence of HR-HPV for ISCC was 96.86%. As expected, HPV-16 was the most common genotype in the 223 ISCC, with the rate of 75.78% (169/223). The second most common genotype was HPV-18, which was detected in 23 (10.31%) ISCC. HPV-58 was the third most common genotype and was detected in 22 (9.87%) ISCC.

### 3.3. HPV Prevalence according to Age Group

**Figure 1:**
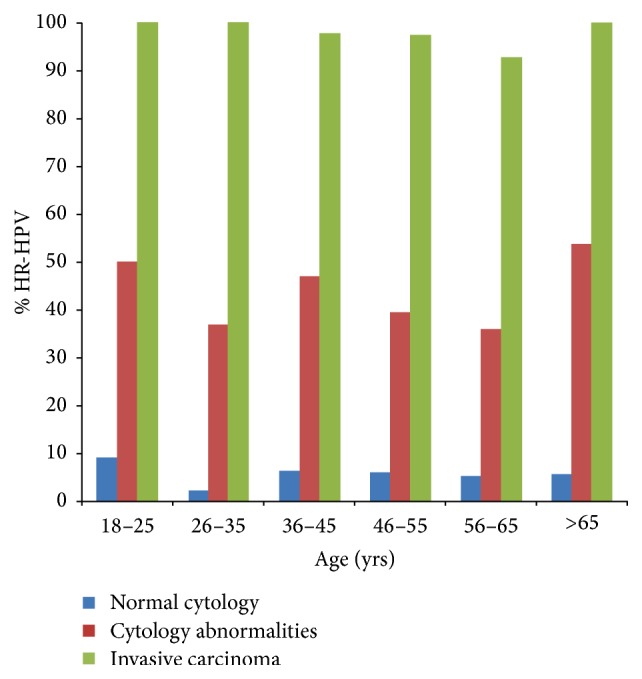
Age-specific prevalence of human papillomavirus in women with normal and abnormal cervical cytology and invasive squamous cell carcinoma.

The 39738 women were classified by their age into 6 groups, and the age-specific prevalence of HR-HPV types was shown in Figure 1. A ripple-shaped age-specific prevalence curve of HR-HPV was observed in women with cytology abnormalities; prevalence decreased from 50% at age 18–25 to a peak of 36.89% at age 26–35 and 46.91% at age 36–45; then prevalence decreased slightly from 39.17% at 46–55 years to 35.71% at ages 56–65 and finally increased up to 53.85% at age >65. No obvious variation has been obtained in ISCC.

## 4. Discussion

Accurate epidemiological information on HPV infections, including genotype-specific prevalence, is essential for achieving further progress in prevention, such as evaluation of potential impact of HPV vaccines, and type-specific monitoring. Therefore, validation set with large size of sample is desired to confirm HPV type in special region because of geographic diversity. To our knowledge, this is the first validation set to comprehensively describe prevalence of HPV infection and its subtypes from normal cytology and cervical cytological abnormalities to carcinoma in a large-scale population of Shantou, which is a high-incidence area of esophageal squamous cell carcinoma.

In the HPV-positive population, HPV-52 genotype was the most common (24.52%), followed by HPV-16 (21.76%) and HPV-58 (14.58%); this present result confirmed our previous findings, which demonstrated that HPV-52 (21.60%), HPV-16 (19.49%), and HPV-58 (13.79%) were the three most prevalent types in overall population. Furthermore, the top three HPV types were consistent with those in Chaozhou's population, which was close to Shantou in terms of geographic location and population genetic background and also named Chaoshan area, with HPV-52 (21.60%), HPV-16 (20.95%), and HPV-58 (15.93%) [11]. Regarding other region-based data in Guangdong province, HPV-16 (20.2%), HPV-52 (17.1%), and HPV-58 (13.2%) were responsible for the three most common types in Pearl River Delta region [12], whereas HPV-16, HPV-58, and HPV-33 were the top HPV types in Shenzhen [13]. Those comparisons suggested that the epidemiological characters of HPV in Chaoshan area were different from other surrounding areas in Guangdong province. Interestingly, Chaoshan was a high-incidence area of esophageal cancer in China [14]. Furthermore, HPV-52 as the highest type in Thai women has been reported and even was common in Asians [15]. Recently, a preliminary study suggested that high attribution of disease to HPV-52 in Asia was due to the high prevalence of lineage B named “Asian lineage” [16].

Overall, the HPV infection occurred in 43.10% of women with cervical cytological abnormalities, of which HR-HPV was the highest in HSIL (56.17%), followed by LSIL (51.64%), ASC-H (48.32%), and ASCU-US (29.69%), and HR-HPV was greater in SIL (54.17%, 299/552) than in ASC (33.21%, 262/789, *P* < 0.001). Although the change trend in this present study was similar to our previous study [6], however, the positive rate was higher in our previous study, for example, 76.64% of SIL and 40.38% of ASC. With restriction to those with HR-HPV, the most prevalent HPV type was HPV-16 (16.85%, 226/1341) in all of the cervical cytology abnormalities, followed by HPV-52 (9.55%, 128/1341) and HPV-58 (7.83%, 105/1341). Similar trends were seen in a previous study of 69 women with cervical cytological abnormalities which indicated that HPV-16, HPV-52, and HPV-58 had the highest frequencies in 213 women with positive HR-HPV [11]. However, the three most frequently detected HR-HPV types were HPV-58 (14.12%), HPV-16 (13.72%), and HPV-52 (12.72%) in cervical abnormalities in our previous study [6]. One possible explanation for this inconsistency is the proportion of women with cervical cytological abnormalities, and this present study (1341) has more samples than the previous study (503). HPV-52 and HPV-58 are more dominant in cervical precancerous lesions in Asia compared to those reported in western countries [17]. For each cervical cytology abnormality, HPV-16 had the highest frequencies in ASC-H (24.16%, 36/149) and HSIL (35.71%, 110/308), while HPV-52 was the most common type in ASC-US (8.28%, 53/640) and LSIL (16.80%, 41/244). Compared with the previous study, only HPV-58 detected as the most common type in LSIL was different. Therefore, the most common types in ASC-H, ASC-US, and HSIL have been confirmed.

We found 96.86% prevalence of HR-HPV in ISCC, which was consistent with 91.8–100% in China [18]. The three most frequent HPV types were HPV-16 (75.78%), HPV-18 (10.31%), and HPV-58 (9.87%). Comparing with the HPV prevalence elsewhere in China, the most common HPV types were HPV-16 (50.6%), followed by HPV-58 (12.4%), HPV-52 (10.9%), and HPV-18 (7.3%) in a large cohort with 1336 invasive cervical cancer cases in Wuhan [19], and HPV-16 (61.2%), followed by HPV-18 (17.7%) and HPV-52 (14.7%) in Hong Kong [20]. Regarding the worldwide prevalence, HPV-18 is the second most carcinogenic HPV type after HPV-16 and accounts for approximately 12% of squamous cell carcinomas worldwide [21]. HPV-16 and HPV-18 comprised the most common viral types in cervical cancer, which are together responsible for approximately 70% of cervical cancers. The most prevalent types in Chinese women were HPV-16, HPV-52, and HPV-58, while HPV-18 was excluded from the top three types. The available vaccines for HPV-18 are therefore expected to have a substantial impact on reducing the burden of cervical cancer in Shantou, even though HPV-18 showed a lower frequency in overall population.

Consequently, our results could be baseline data and validation set, which provide the most robust available estimates of the prevalence of type-specific HPV, in normal cytological and cervical cytological abnormalities and cervical cancer, prior to HPV immunization in Shantou's population.

## Figures and Tables

**Table 1:** Human HPV prevalence in overall population and women with normal cytology.

Positive	Total *n* = 39738 (%)	Women with normal cytology *n* = 38397 (%)
HPV	3300 (8.30 )	2722 (7.09)
HR-HPV	3300 (8.30 )	2499 (6.51)
LR-HPV	444 (1.12)	397 (1.03)
Single HPV	2530 (6.37)	2105 (5.48)
Multiple HPV	770 (1.94)	617 (1.61)
Multiple HR-HPV	653 (1.64)	510 (1.33)

**Table 2:** Human HPV type-specific distribution in overall population and women with normal cytology.

Types	Total			Women with normal cytology		
	*N*	% of all (*n* = 39738)	% of HPV-positive (*n* = 3300)	*N*	% of all (*n* = 38397)	% of HPV-positive (*n* = 2722)
*High risk*						
16	718	1.81	21.76	492	1.28	18.07
18	280	0.70	8.48	220	0.57	8.08
31	142	0.36	4.30	113	0.29	4.15
33	212	0.53	6.42	159	0.41	5.84
35	64	0.16	1.94	48	0.12	1.76
39	160	0.40	4.85	142	0.37	5.22
45	67	0.17	2.03	57	0.15	2.09
51	60	0.15	1.82	51	0.13	1.87
52	809	2.04	24.52	681	1.76	25.02
53	359	0.90	10.88	313	0.81	11.50
56	82	0.21	2.48	70	0.18	2.57
58	481	1.21	14.58	376	0.97	13.81
59	104	0.26	3.15	93	0.24	3.42
66	145	0.36	4.39	127	0.33	4.67
68	230	0.58	6.97	203	0.52	7.46
*Low risk*						
6	189	0.48	5.73	173	0.45	6.36
11	177	0.45	5.36	152	0.39	5.58
42	30	0.08	0.91	27	0.07	0.99
43	6	0.02	0.18	6	0.02	0.22
44	60	0.15	1.82	55	0.14	2.02

**Table 3:** Human HPV prevalence in women with cervical cytology abnormalities and ISCC.

Positive	ASC-H (*n* = 149)	ASC-US (*n* = 640)	HSIL (*n* = 308)	LSIL (*n* = 244)	ISCC (*n* = 223)
HPV	73 (48.99)	201 (31.41)	175 (56.8)	129 (52.87)	216 (96.86)
HR-HPV	72 (48.32)	190 (29.69)	173 (56.1)	126 (51.64)	216 (96.86)
LR- HPV	5 (3.36)	27 (4.22)	5 (1.62)	10 (4.10)	2 (0.90)
Single HPV	54 (36.24)	138 (21.56)	142 (46.10)	91 (37.30)	182 (81.61)
Multiple HPV	19 (12.75)	63 (9.84)	33 (10.71)	38 (15.57)	34 (15.25)
Multiple HR-HPV	16 (10.74)	59 (9.22)	33 (10.71)	35 (14.34)	34 (15.25)

**Table 4:** Human papillomavirus type-specific distribution in women with cervical cytology abnormalities and ISCC.

	ASCH (*n* = 149)	ASC-US (*n* = 640)	HSIL (*n* = 308)	LSIL (*n* = 244)	ISCC (*n* = 223)
*High risk*					
16	36 (24.16)	49 (7.66)	110 (35.71)	31 (12.70)	169 (75.78)
18	7 (4.70)	25 (3.91)	18 (5.84)	10 (4.10)	23 (10.31)
31	0 (0.00)	15 (2.34)	7 (2.27)	7 (2.87)	4 (1.79)
33	7 (4.70)	20 (3.13)	14 (4.55)	12 (4.92)	11 (4.93)
35	2 (1.34)	7 (1.09)	1 (0.32)	6 (2.46)	0 (0.00)
39	1 (0.67)	10 (1.56)	3 (0.97)	4 (1.64)	5 (2.24)
45	1 (0.67)	3 (0.47)	2 (0.65)	4 (1.64)	3 (1.35)
51	1 (0.67)	3 (0.47)	1 (0.32)	4 (1.64)	2 (0.90)
52	18 (12.08)	53 (8.28)	16 (5.19)	41 (16.80)	15 (6.73)
53	1 (0.67)	25 (3.91)	5 (1.62)	15 (6.15)	1 (0.45)
56	0 (0.00)	7 (1.09)	2 (0.65)	3 (1.23)	1 (0.45)
58	16 (10.74)	38 (5.94)	28 (9.09)	23 (9.43)	22 (9.87)
59	0 (0.00)	5 (0.78)	2 (0.65)	4 (1.64)	4 (1.79)
66	1 (0.67)	11 (1.72)	3 (0.97)	3 (1.23)	0 (0.00)
68	3 (2.01)	11 (1.72)	3 (0.97)	10 (4.10)	2 (0.90)
*Low risk*					
6	2 (1.34)	11 (1.72)	0 (0.00)	3 (1.23)	0 (0.00)
11	2 (1.34)	14 (2.19)	4 (1.30)	5 (2.05)	1 (0.45)
42	1 (0.67)	1 (0.16)	1 (0.32)	0 (0.00)	0 (0.00)
43	0 (0.00)	0 (0.00)	0 (0.00)	0 (0.00)	0 (0.00)
44	0 (0.00)	2 (0.31)	0 (0.00)	3 (1.23)	1 (0.45)
